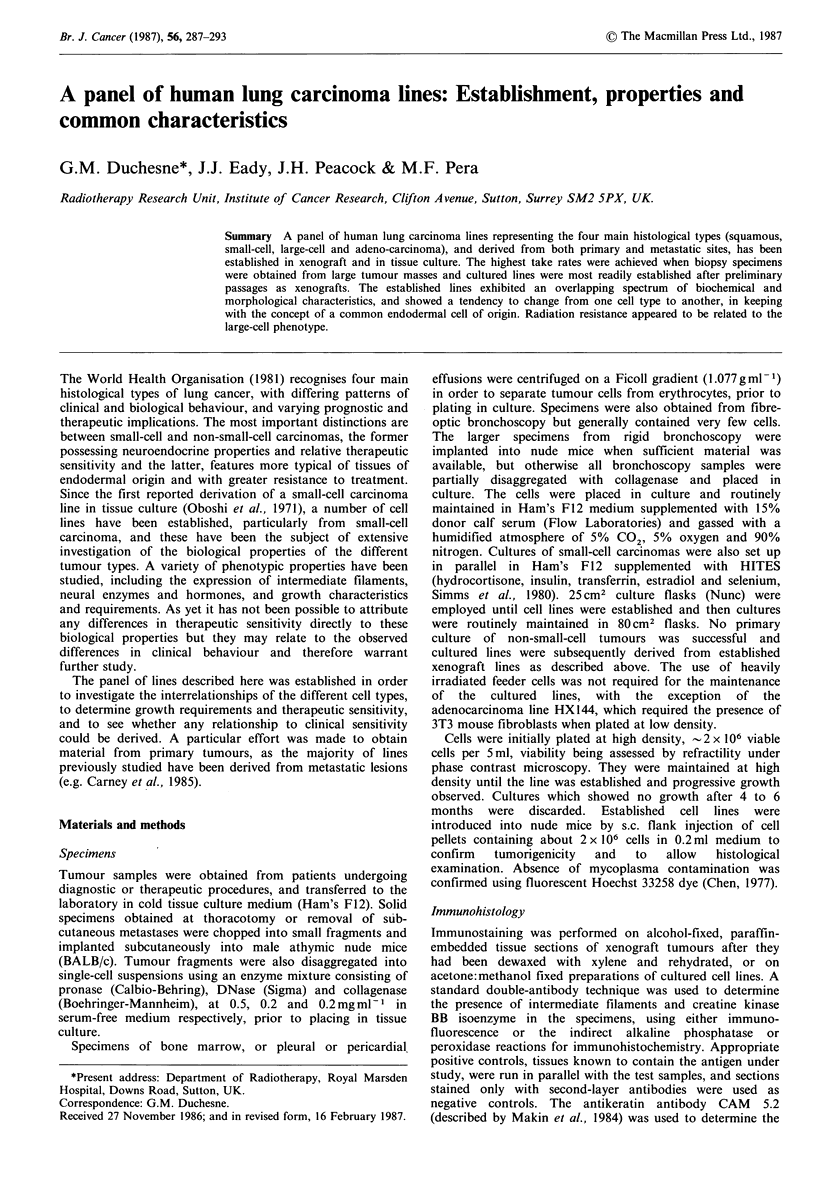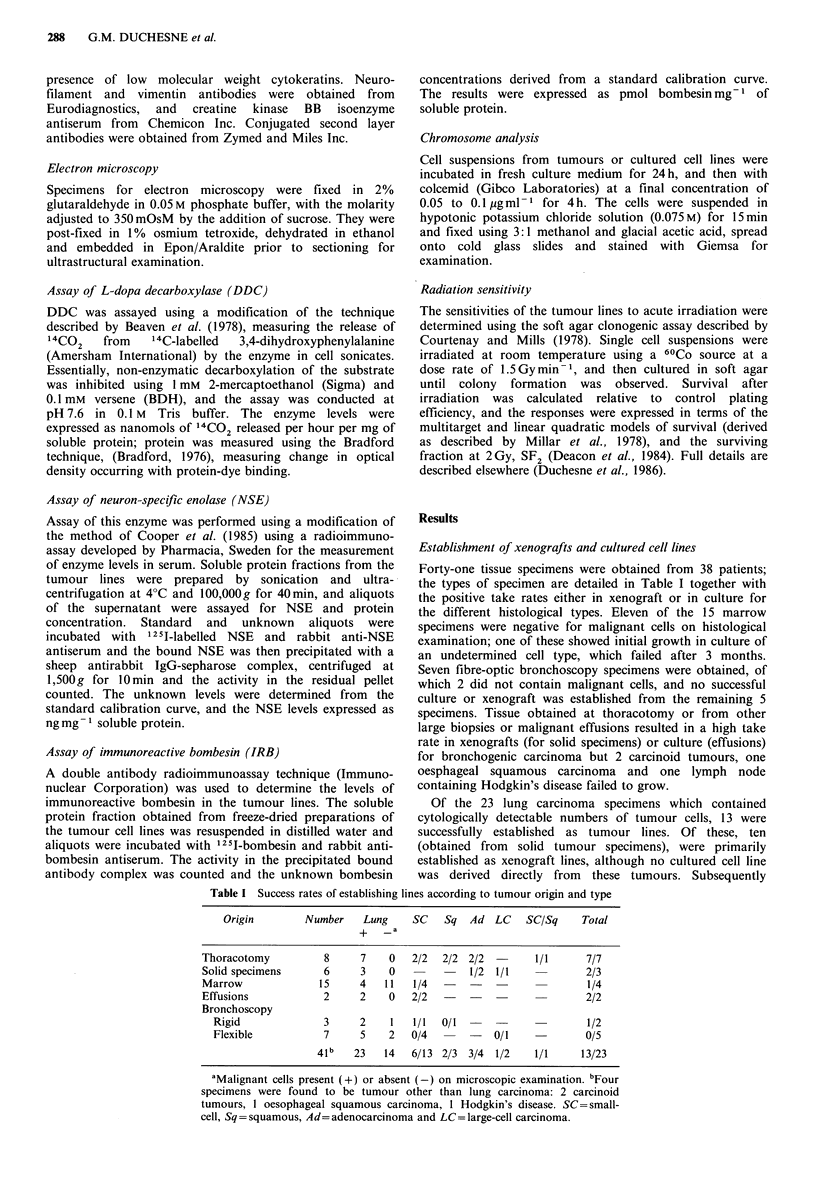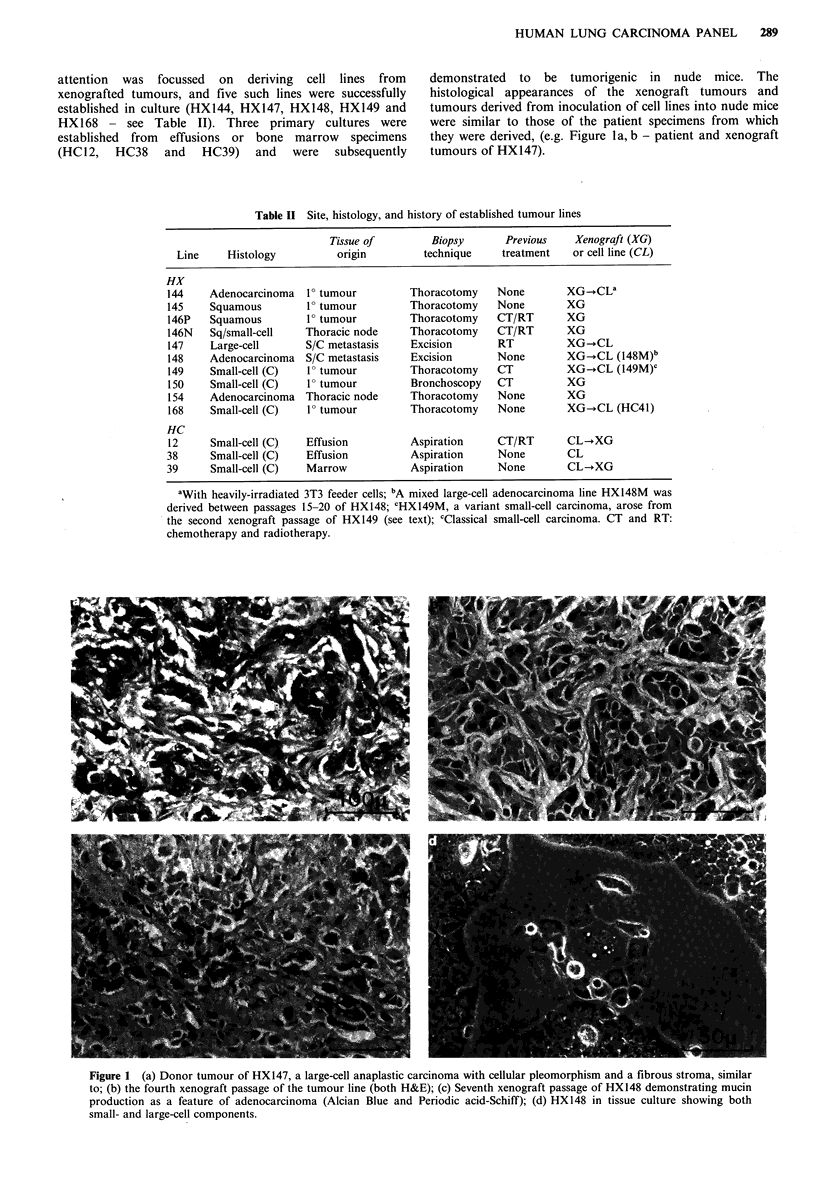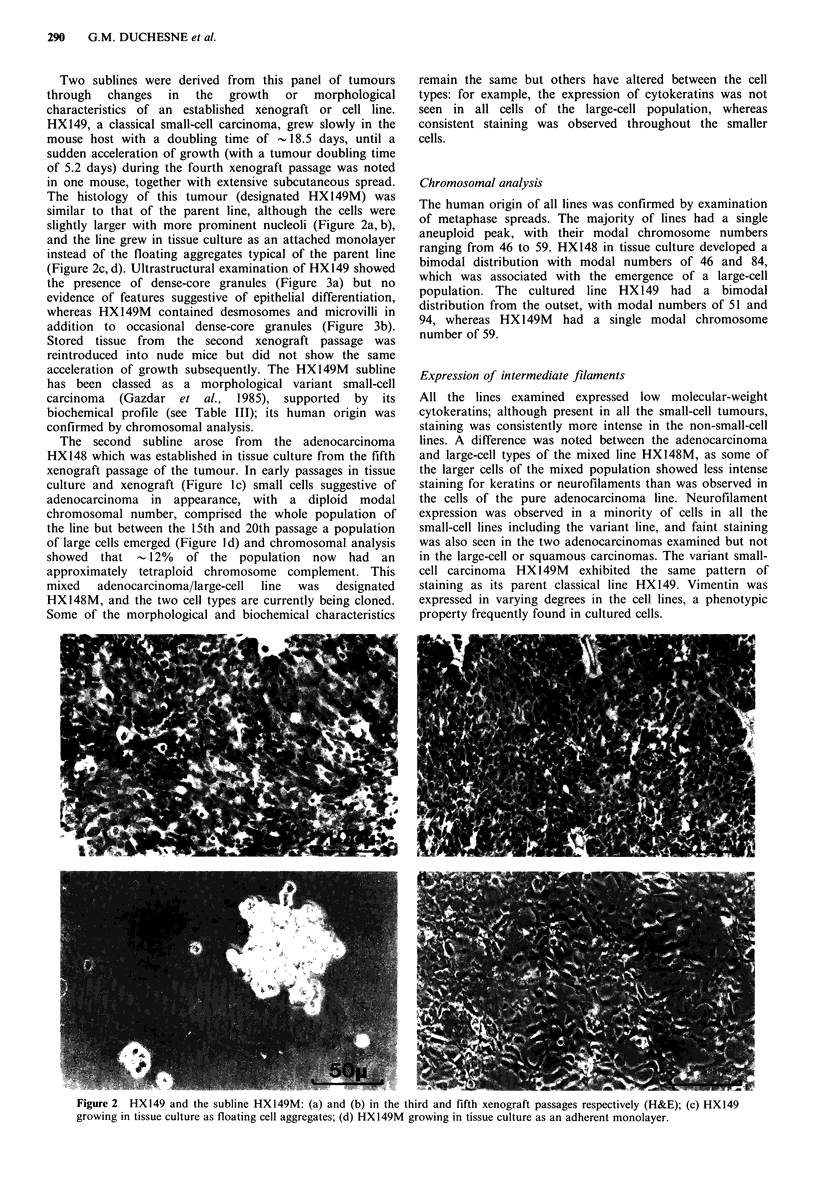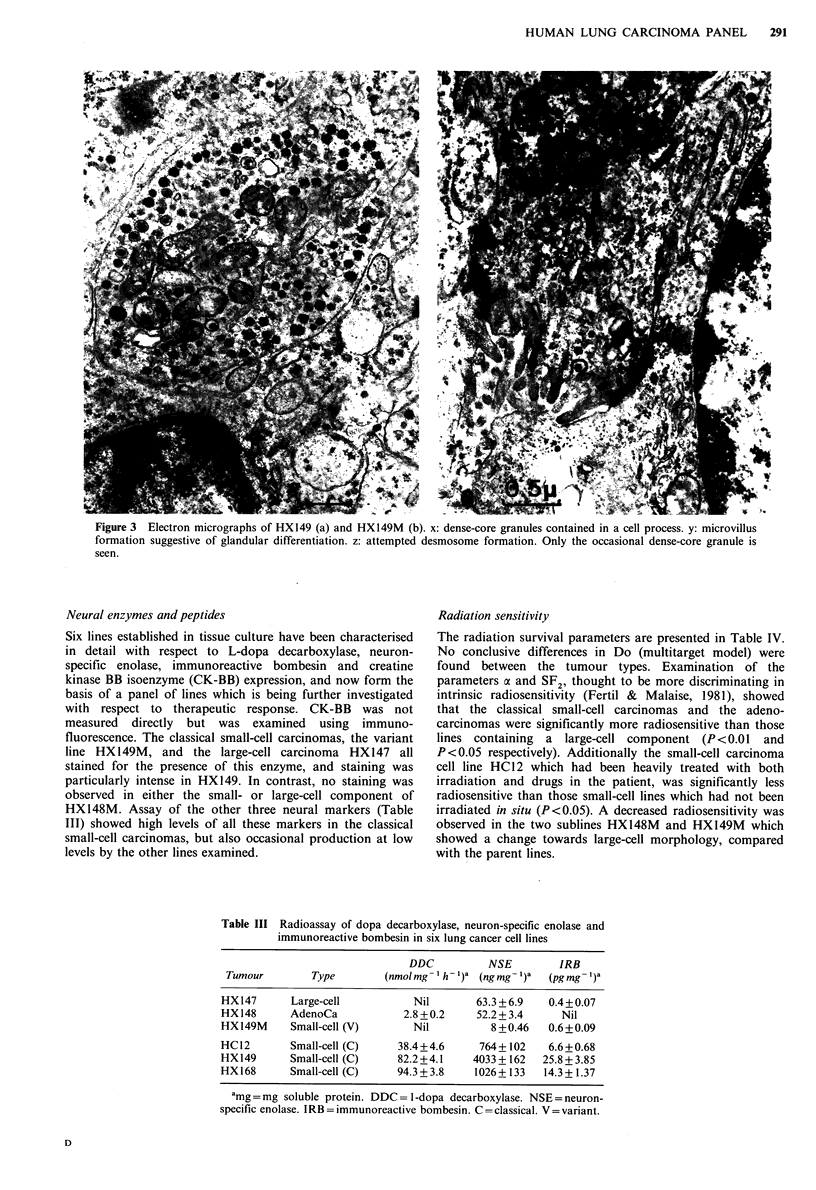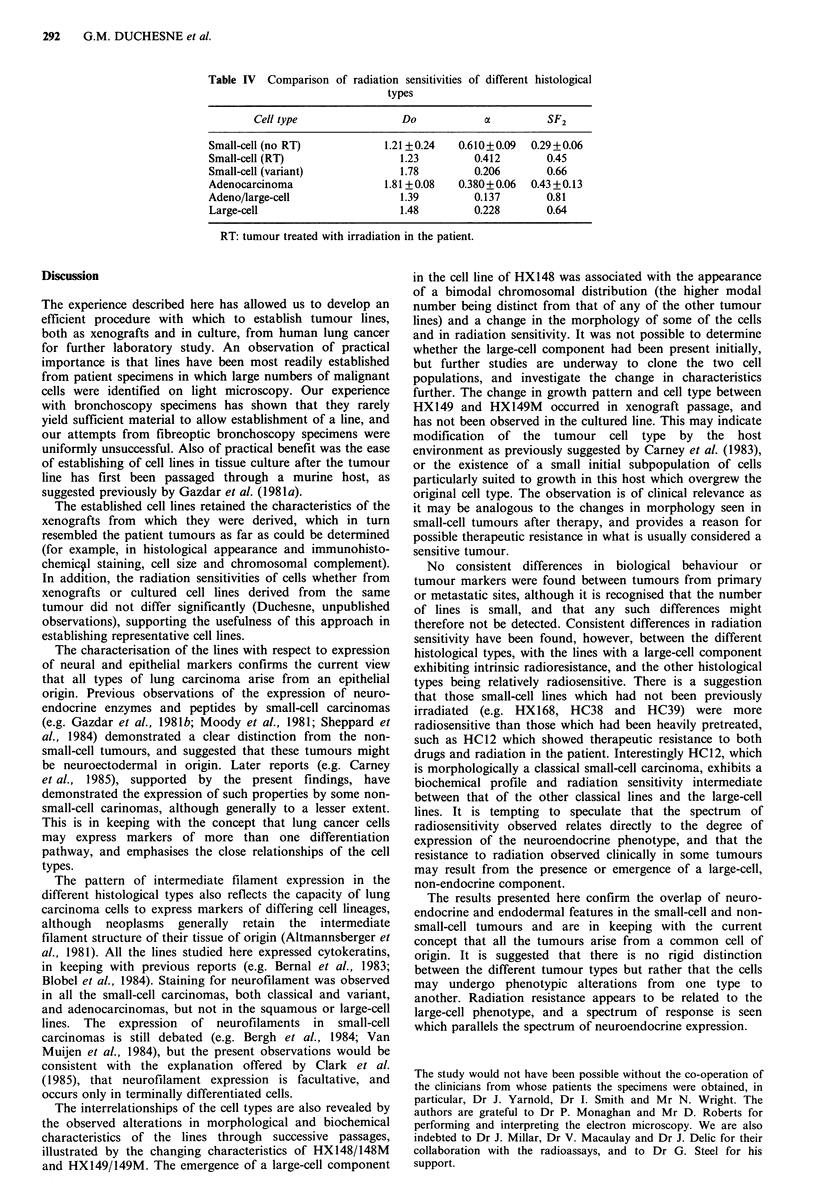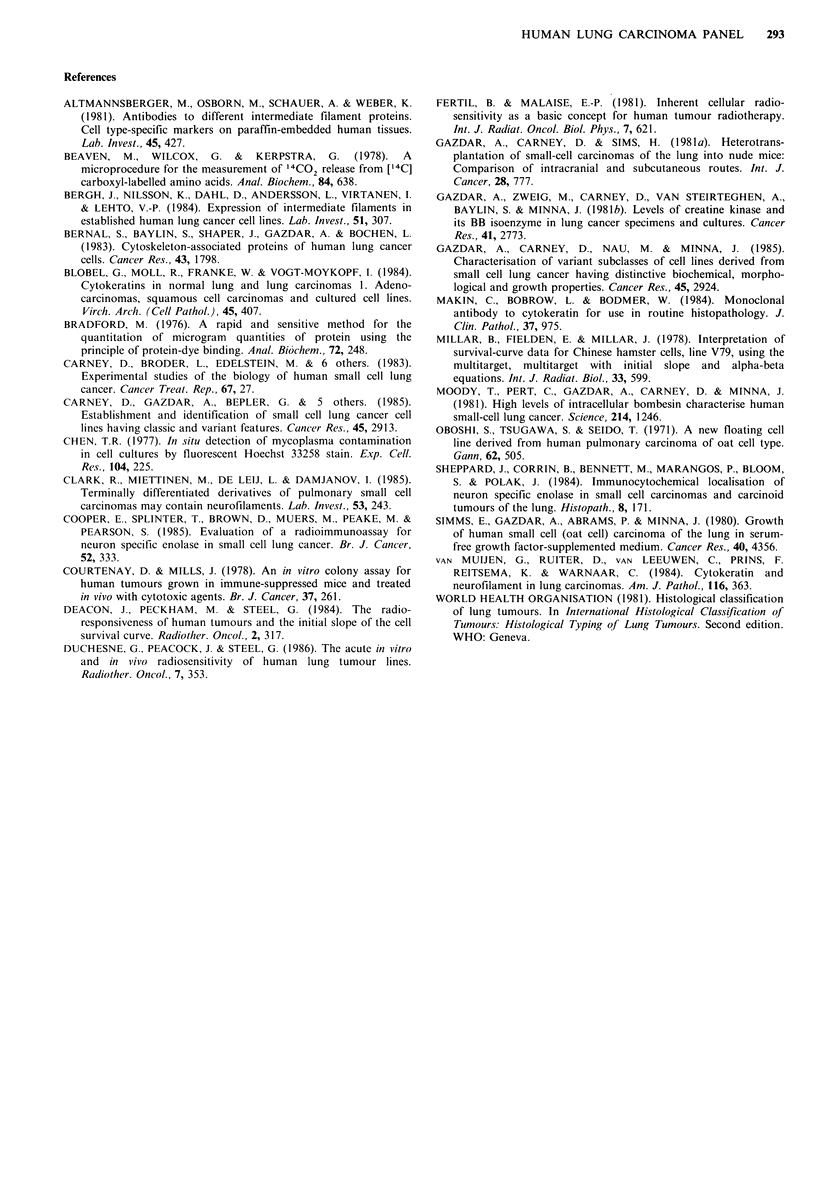# A panel of human lung carcinoma lines: establishment, properties and common characteristics.

**DOI:** 10.1038/bjc.1987.191

**Published:** 1987-09

**Authors:** G. M. Duchesne, J. J. Eady, J. H. Peacock, M. F. Pera

**Affiliations:** Radiotherapy Research Unit, Institute of Cancer Research, Sutton, Surrey, UK.

## Abstract

**Images:**


Br. J. Cancer (1987), 56, 287–293 © The Macmillan Press Ltd., 1987

### A panel of human lung carcinoma lines: Establishment, properties and common characteristics

G.M. Duchesne*, J.J. Eady, J.H. Peacock & M.F. Pera

*Radiotherapy Research Unit, Institute of Cancer Research, Clifton Avenue, Sutton, Surrey SM2 5PX, UK.*

**Summary** A panel of human lung carcinoma lines representing the four main histological types (squamous, small-cell, large-cell and adeno-carcinoma), and derived from both primary and metastatic sites, has been established in xenograft and in tissue culture. The highest take rates were achieved when biopsy specimens were obtained from large tumour masses and cultured lines were most readily established after preliminary passages as xenografts. The established lines exhibited an overlapping spectrum of biochemical and morphological characteristics, and showed a tendency to change from one cell type to another, in keeping with the concept of a common endodermal cell of origin. Radiation resistance appeared to be related to the large-cell phenotype.

The World Health Organisation (1981) recognises four main histological types of lung cancer, with differing patterns of clinical and biological behaviour, and varying prognostic and therapeutic implications. The most important distinctions are between small-cell and non-small-cell carcinomas, the former possessing neuroendocrine properties and relative therapeutic sensitivity and the latter, features more typical of tissues of endodermal origin and with greater resistance to treatment. Since the first reported derivation of a small-cell carcinoma line in tissue culture (Oboshi *et al.*, 1971), a number of cell lines have been established, particularly from small-cell carcinoma, and these have been the subject of extensive investigation of the biological properties of the different tumour types. A variety of phenotypic properties have been studied, including the expression of intermediate filaments, neural enzymes and hormones, and growth characteristics and requirements. As yet it has not been possible to attribute any differences in therapeutic sensitivity directly to these biological properties but they may relate to the observed differences in clinical behaviour and therefore warrant further study.

The panel of lines described here was established in order to investigate the interrelationships of the different cell types, to determine growth requirements and therapeutic sensitivity, and to see whether any relationship to clinical sensitivity could be derived. A particular effort was made to obtain material from primary tumours, as the majority of lines previously studied have been derived from metastatic lesions (e.g. Carney *et al.*, 1985).

### Materials and methods

### *Specimens*

Tumour samples were obtained from patients undergoing diagnostic or therapeutic procedures, and transferred to the laboratory in cold tissue culture medium (Ham's F12). Solid specimens obtained at thoracotomy or removal of subcutaneous metastases were chopped into small fragments and implanted subcutaneously into male athymic nude mice (BALB/c). Tumour fragments were also disaggregated into single-cell suspensions using an enzyme mixture consisting of pronase (Calbio-Behring), DNase (Sigma) and collagenase (Boehringer-Mannheim), at 0.5, 0.2 and 0.2 $mg\,ml^{-1}$ in serum-free medium respectively, prior to placing in tissue culture.

Specimens of bone marrow, or pleural or pericardial effusions were centrifuged on a Ficoll gradient (1.077 $g\,ml^{-1}$) in order to separate tumour cells from erythrocytes, prior to plating in culture. Specimens were also obtained from fibreoptic bronchoscopy but generally contained very few cells. The larger specimens from rigid bronchoscopy were implanted into nude mice when sufficient material was available, but otherwise all bronchoscopy samples were partially disaggregated with collagenase and placed in culture. The cells were placed in culture and routinely maintained in Ham's F12 medium supplemented with 15% donor calf serum (Flow Laboratories) and gassed with a humidified atmosphere of 5% $CO_2$, 5% oxygen and 90% nitrogen. Cultures of small-cell carcinomas were also set up in parallel in Ham's F12 supplemented with HITES (hydrocortisone, insulin, transferrin, estradiol and selenium, Simms *et al.*, 1980). 25 $cm^2$ culture flasks (Nunc) were employed until cell lines were established and then cultures were routinely maintained in 80 $cm^2$ flasks. No primary culture of non-small-cell tumours was successful and cultured lines were subsequently derived from established xenograft lines as described above. The use of heavily irradiated feeder cells was not required for the maintenance of the cultured lines, with the exception of the adenocarcinoma line HX144, which required the presence of 3T3 mouse fibroblasts when plated at low density.

Cells were initially plated at high density, $\sim 2\times10^6$ viable cells per 5 ml, viability being assessed by refractility under phase contrast microscopy. They were maintained at high density until the line was established and progressive growth observed. Cultures which showed no growth after 4 to 6 months were discarded. Established cell lines were introduced into nude mice by s.c. flank injection of cell pellets containing about $2\times10^6$ cells in 0.2 ml medium to confirm tumorigenicity and to allow histological examination. Absence of mycoplasma contamination was confirmed using fluorescent Hoechst 33258 dye (Chen, 1977).

### *Immunohistology*

Immunostaining was performed on alcohol-fixed, paraffin-embedded tissue sections of xenograft tumours after they had been dewaxed with xylene and rehydrated, or on acetone:methanol fixed preparations of cultured cell lines. A standard double-antibody technique was used to determine the presence of intermediate filaments and creatine kinase BB isoenzyme in the specimens, using either immunofluorescence or the indirect alkaline phosphatase or peroxidase reactions for immunohistochemistry. Appropriate positive controls, tissues known to contain the antigen under study, were run in parallel with the test samples, and sections stained only with second-layer antibodies were used as negative controls. The antikeratin antibody CAM 5.2 (described by Makin *et al.*, 1984) was used to determine the

*Present address: Department of Radiotherapy, Royal Marsden Hospital, Downs Road, Sutton, UK.
Correspondence: G.M. Duchesne.
Received 27 November 1986; and in revised form, 16 February 1987.

presence of low molecular weight cytokeratins. Neurofilament and vimentin antibodies were obtained from Eurodiagnostics, and creatine kinase BB isoenzyme antiserum from Chemicon Inc. Conjugated second layer antibodies were obtained from Zymed and Miles Inc.

*Electron microscopy*

Specimens for electron microscopy were fixed in 2% glutaraldehyde in 0.05 M phosphate buffer, with the molarity adjusted to 350 mOsM by the addition of sucrose. They were post-fixed in 1% osmium tetroxide, dehydrated in ethanol and embedded in Epon/Araldite prior to sectioning for ultrastructural examination.

*Assay of L-dopa decarboxylase (DDC)*

DDC was assayed using a modification of the technique described by Beaven *et al.* (1978), measuring the release of $^{14}CO_2$ from $^{14}C$-labelled 3,4-dihydroxyphenylalanine (Amersham International) by the enzyme in cell sonicates. Essentially, non-enzymatic decarboxylation of the substrate was inhibited using 1 mM 2-mercaptoethanol (Sigma) and 0.1 mM versene (BDH), and the assay was conducted at pH 7.6 in 0.1 M Tris buffer. The enzyme levels were expressed as nanomols of $^{14}CO_2$ released per hour per mg of soluble protein; protein was measured using the Bradford technique, (Bradford, 1976), measuring change in optical density occurring with protein-dye binding.

*Assay of neuron-specific enolase (NSE)*

Assay of this enzyme was performed using a modification of the method of Cooper *et al.* (1985) using a radioimmunoassay developed by Pharmacia, Sweden for the measurement of enzyme levels in serum. Soluble protein fractions from the tumour lines were prepared by sonication and ultracentrifugation at 4°C and 100,000 *g* for 40 min, and aliquots of the supernatant were assayed for NSE and protein concentration. Standard and unknown aliquots were incubated with $^{125}I$-labelled NSE and rabbit anti-NSE antiserum and the bound NSE was then precipitated with a sheep antirabbit IgG-sepharose complex, centrifuged at 1,500 *g* for 10 min and the activity in the residual pellet counted. The unknown levels were determined from the standard calibration curve, and the NSE levels expressed as $ng\,mg^{-1}$ soluble protein.

*Assay of immunoreactive bombesin (IRB)*

A double antibody radioimmunoassay technique (Immunonuclear Corporation) was used to determine the levels of immunoreactive bombesin in the tumour lines. The soluble protein fraction obtained from freeze-dried preparations of the tumour cell lines was resuspended in distilled water and aliquots were incubated with $^{125}I$-bombesin and rabbit anti-bombesin antiserum. The activity in the precipitated bound antibody complex was counted and the unknown bombesin concentrations derived from a standard calibration curve. The results were expressed as pmol bombesin $mg^{-1}$ of soluble protein.

*Chromosome analysis*

Cell suspensions from tumours or cultured cell lines were incubated in fresh culture medium for 24 h, and then with colcemid (Gibco Laboratories) at a final concentration of 0.05 to 0.1 $\mu g\,ml^{-1}$ for 4 h. The cells were suspended in hypotonic potassium chloride solution (0.075 M) for 15 min and fixed using 3:1 methanol and glacial acetic acid, spread onto cold glass slides and stained with Giemsa for examination.

*Radiation sensitivity*

The sensitivities of the tumour lines to acute irradiation were determined using the soft agar clonogenic assay described by Courtenay and Mills (1978). Single cell suspensions were irradiated at room temperature using a $^{60}Co$ source at a dose rate of 1.5 Gy $min^{-1}$, and then cultured in soft agar until colony formation was observed. Survival after irradiation was calculated relative to control plating efficiency, and the responses were expressed in terms of the multitarget and linear quadratic models of survival (derived as described by Millar *et al.*, 1978), and the surviving fraction at 2 Gy, $SF_2$ (Deacon *et al.*, 1984). Full details are described elsewhere (Duchesne *et al.*, 1986).

### Results

*Establishment of xenografts and cultured cell lines*

Forty-one tissue specimens were obtained from 38 patients; the types of specimen are detailed in Table I together with the positive take rates either in xenograft or in culture for the different histological types. Eleven of the 15 marrow specimens were negative for malignant cells on histological examination; one of these showed initial growth in culture of an undetermined cell type, which failed after 3 months. Seven fibre-optic bronchoscopy specimens were obtained, of which 2 did not contain malignant cells, and no successful culture or xenograft was established from the remaining 5 specimens. Tissue obtained at thoracotomy or from other large biopsies or malignant effusions resulted in a high take rate in xenografts (for solid specimens) or culture (effusions) for bronchogenic carcinoma but 2 carcinoid tumours, one oesphageal squamous carcinoma and one lymph node containing Hodgkin's disease failed to grow.

Of the 23 lung carcinoma specimens which contained cytologically detectable numbers of tumour cells, 13 were successfully established as tumour lines. Of these, ten (obtained from solid tumour specimens), were primarily established as xenograft lines, although no cultured cell line was derived directly from these tumours. Subsequently

**Table I** Success rates of establishing lines according to tumour origin and type

| *Origin* | *Number* | *Lung* + | −[a] | *SC* | *Sq* | *Ad* | *LC* | *SC/Sq* | *Total* |
|---|---|---|---|---|---|---|---|---|---|
| Thoracotomy | 8 | 7 | 0 | 2/2 | 2/2 | 2/2 | — | 1/1 | 7/7 |
| Solid specimens | 6 | 3 | 0 | — | — | 1/2 | 1/1 | — | 2/3 |
| Marrow | 15 | 4 | 11 | 1/4 | — | — | — | — | 1/4 |
| Effusions | 2 | 2 | 0 | 2/2 | — | — | — | — | 2/2 |
| Bronchoscopy | | | | | | | | | |
| Rigid | 3 | 2 | 1 | 1/1 | 0/1 | — | — | — | 1/2 |
| Flexible | 7 | 5 | 2 | 0/4 | — | — | 0/1 | — | 0/5 |
| | 41[b] | 23 | 14 | 6/13 | 2/3 | 3/4 | 1/2 | 1/1 | 13/23 |

[a]Malignant cells present (+) or absent (−) on microscopic examination. [b]Four specimens were found to be tumour other than lung carcinoma: 2 carcinoid tumours, 1 oesophageal squamous carcinoma, 1 Hodgkin's disease. *SC* = small-cell, *Sq* = squamous, *Ad* = adenocarcinoma and *LC* = large-cell carcinoma.

attention was focussed on deriving cell lines from xenografted tumours, and five such lines were successfully established in culture (HX144, HX147, HX148, HX149 and HX168 – see Table II). Three primary cultures were established from effusions or bone marrow specimens (HC12, HC38 and HC39) and were subsequently demonstrated to be tumorigenic in nude mice. The histological appearances of the xenograft tumours and tumours derived from inoculation of cell lines into nude mice were similar to those of the patient specimens from which they were derived, (e.g. Figure 1a, b – patient and xenograft tumours of HX147).

**Table II** Site, histology, and history of established tumour lines

| Line | Histology | *Tissue of* origin | *Biopsy* technique | *Previous* treatment | *Xenograft (XG)* or cell line (*CL*) |
|---|---|---|---|---|---|
| *HX* | | | | | |
| 144 | Adenocarcinoma | 1° tumour | Thoracotomy | None | XG→CL[a] |
| 145 | Squamous | 1° tumour | Thoracotomy | None | XG |
| 146P | Squamous | 1° tumour | Thoracotomy | CT/RT | XG |
| 146N | Sq/small-cell | Thoracic node | Thoracotomy | CT/RT | XG |
| 147 | Large-cell | S/C metastasis | Excision | RT | XG→CL |
| 148 | Adenocarcinoma | S/C metastasis | Excision | None | XG→CL (148M)[b] |
| 149 | Small-cell (C) | 1° tumour | Thoracotomy | CT | XG→CL (149M)[c] |
| 150 | Small-cell (C) | 1° tumour | Bronchoscopy | CT | XG |
| 154 | Adenocarcinoma | Thoracic node | Thoracotomy | None | XG |
| 168 | Small-cell (C) | 1° tumour | Thoracotomy | None | XG→CL (HC41) |
| *HC* | | | | | |
| 12 | Small-cell (C) | Effusion | Aspiration | CT/RT | CL→XG |
| 38 | Small-cell (C) | Effusion | Aspiration | None | CL |
| 39 | Small-cell (C) | Marrow | Aspiration | None | CL→XG |

[a]With heavily-irradiated 3T3 feeder cells; [b]A mixed large-cell adenocarcinoma line HX148M was derived between passages 15–20 of HX148; [c]HX149M, a variant small-cell carcinoma, arose from the second xenograft passage of HX149 (see text); [c]Classical small-cell carcinoma. CT and RT: chemotherapy and radiotherapy.

**Figure 1** (a) Donor tumour of HX147, a large-cell anaplastic carcinoma with cellular pleomorphism and a fibrous stroma, similar to; (b) the fourth xenograft passage of the tumour line (both H&E); (c) Seventh xenograft passage of HX148 demonstrating mucin production as a feature of adenocarcinoma (Alcian Blue and Periodic acid-Schiff); (d) HX148 in tissue culture showing both small- and large-cell components.

Two sublines were derived from this panel of tumours through changes in the growth or morphological characteristics of an established xenograft or cell line. HX149, a classical small-cell carcinoma, grew slowly in the mouse host with a doubling time of ~18.5 days, until a sudden acceleration of growth (with a tumour doubling time of 5.2 days) during the fourth xenograft passage was noted in one mouse, together with extensive subcutaneous spread. The histology of this tumour (designated HX149M) was similar to that of the parent line, although the cells were slightly larger with more prominent nucleoli (Figure 2a, b), and the line grew in tissue culture as an attached monolayer instead of the floating aggregates typical of the parent line (Figure 2c, d). Ultrastructural examination of HX149 showed the presence of dense-core granules (Figure 3a) but no evidence of features suggestive of epithelial differentiation, whereas HX149M contained desmosomes and microvilli in addition to occasional dense-core granules (Figure 3b). Stored tissue from the second xenograft passage was reintroduced into nude mice but did not show the same acceleration of growth subsequently. The HX149M subline has been classed as a morphological variant small-cell carcinoma (Gazdar *et al.*, 1985), supported by its biochemical profile (see Table III); its human origin was confirmed by chromosomal analysis.

The second subline arose from the adenocarcinoma HX148 which was established in tissue culture from the fifth xenograft passage of the tumour. In early passages in tissue culture and xenograft (Figure 1c) small cells suggestive of adenocarcinoma in appearance, with a diploid modal chromosomal number, comprised the whole population of the line but between the 15th and 20th passage a population of large cells emerged (Figure 1d) and chromosomal analysis showed that ~12% of the population now had an approximately tetraploid chromosome complement. This mixed adenocarcinoma/large-cell line was designated HX148M, and the two cell types are currently being cloned. Some of the morphological and biochemical characteristics remain the same but others have altered between the cell types: for example, the expression of cytokeratins was not seen in all cells of the large-cell population, whereas consistent staining was observed throughout the smaller cells.

### *Chromosomal analysis*

The human origin of all lines was confirmed by examination of metaphase spreads. The majority of lines had a single aneuploid peak, with their modal chromosome numbers ranging from 46 to 59. HX148 in tissue culture developed a bimodal distribution with modal numbers of 46 and 84, which was associated with the emergence of a large-cell population. The cultured line HX149 had a bimodal distribution from the outset, with modal numbers of 51 and 94, whereas HX149M had a single modal chromosome number of 59.

### *Expression of intermediate filaments*

All the lines examined expressed low molecular-weight cytokeratins; although present in all the small-cell tumours, staining was consistently more intense in the non-small-cell lines. A difference was noted between the adenocarcinoma and large-cell types of the mixed line HX148M, as some of the larger cells of the mixed population showed less intense staining for keratins or neurofilaments than was observed in the cells of the pure adenocarcinoma line. Neurofilament expression was observed in a minority of cells in all the small-cell lines including the variant line, and faint staining was also seen in the two adenocarcinomas examined but not in the large-cell or squamous carcinomas. The variant small-cell carcinoma HX149M exhibited the same pattern of staining as its parent classical line HX149. Vimentin was expressed in varying degrees in the cell lines, a phenotypic property frequently found in cultured cells.

**Figure 2** HX149 and the subline HX149M: (a) and (b) in the third and fifth xenograft passages respectively (H&E); (c) HX149 growing in tissue culture as floating cell aggregates; (d) HX149M growing in tissue culture as an adherent monolayer.

**Figure 3** Electron micrographs of HX149 (a) and HX149M (b). x: dense-core granules contained in a cell process. y: microvillus formation suggestive of glandular differentiation. z: attempted desmosome formation. Only the occasional dense-core granule is seen.

*Neural enzymes and peptides*

Six lines established in tissue culture have been characterised in detail with respect to L-dopa decarboxylase, neuron-specific enolase, immunoreactive bombesin and creatine kinase BB isoenzyme (CK-BB) expression, and now form the basis of a panel of lines which is being further investigated with respect to therapeutic response. CK-BB was not measured directly but was examined using immunofluorescence. The classical small-cell carcinomas, the variant line HX149M, and the large-cell carcinoma HX147 all stained for the presence of this enzyme, and staining was particularly intense in HX149. In contrast, no staining was observed in either the small- or large-cell component of HX148M. Assay of the other three neural markers (Table III) showed high levels of all these markers in the classical small-cell carcinomas, but also occasional production at low levels by the other lines examined.

*Radiation sensitivity*

The radiation survival parameters are presented in Table IV. No conclusive differences in Do (multitarget model) were found between the tumour types. Examination of the parameters $\alpha$ and $SF_2$, thought to be more discriminating in intrinsic radiosensitivity (Fertil & Malaise, 1981), showed that the classical small-cell carcinomas and the adenocarcinomas were significantly more radiosensitive than those lines containing a large-cell component ($P<0.01$ and $P<0.05$ respectively). Additionally the small-cell carcinoma cell line HC12 which had been heavily treated with both irradiation and drugs in the patient, was significantly less radiosensitive than those small-cell lines which had not been irradiated *in situ* ($P<0.05$). A decreased radiosensitivity was observed in the two sublines HX148M and HX149M which showed a change towards large-cell morphology, compared with the parent lines.

**Table III** Radioassay of dopa decarboxylase, neuron-specific enolase and immunoreactive bombesin in six lung cancer cell lines

| *Tumour* | *Type* | *DDC* ($nmol\,mg^{-1}\,h^{-1}$)[a] | *NSE* ($ng\,mg^{-1}$)[a] | *IRB* ($pg\,mg^{-1}$)[a] |
|---|---|---|---|---|
| HX147 | Large-cell | Nil | 63.3±6.9 | 0.4±0.07 |
| HX148 | AdenoCa | 2.8±0.2 | 52.2±3.4 | Nil |
| HX149M | Small-cell (V) | Nil | 8±0.46 | 0.6±0.09 |
| HC12 | Small-cell (C) | 38.4±4.6 | 764±102 | 6.6±0.68 |
| HX149 | Small-cell (C) | 82.2±4.1 | 4033±162 | 25.8±3.85 |
| HX168 | Small-cell (C) | 94.3±3.8 | 1026±133 | 14.3±1.37 |

[a]mg = mg soluble protein. DDC = l-dopa decarboxylase. NSE = neuron-specific enolase. IRB = immunoreactive bombesin. C = classical. V = variant.

Table IV Comparison of radiation sensitivities of different histological types

| Cell type | Do | α | $SF_2$ |
|---|---|---|---|
| Small-cell (no RT) | 1.21±0.24 | 0.610±0.09 | 0.29±0.06 |
| Small-cell (RT) | 1.23 | 0.412 | 0.45 |
| Small-cell (variant) | 1.78 | 0.206 | 0.66 |
| Adenocarcinoma | 1.81±0.08 | 0.380±0.06 | 0.43±0.13 |
| Adeno/large-cell | 1.39 | 0.137 | 0.81 |
| Large-cell | 1.48 | 0.228 | 0.64 |

RT: tumour treated with irradiation in the patient.

### Discussion

The experience described here has allowed us to develop an efficient procedure with which to establish tumour lines, both as xenografts and in culture, from human lung cancer for further laboratory study. An observation of practical importance is that lines have been most readily established from patient specimens in which large numbers of malignant cells were identified on light microscopy. Our experience with bronchoscopy specimens has shown that they rarely yield sufficient material to allow establishment of a line, and our attempts from fibreoptic bronchoscopy specimens were uniformly unsuccessful. Also of practical benefit was the ease of establishing of cell lines in tissue culture after the tumour line has first been passaged through a murine host, as suggested previously by Gazdar *et al.* (1981*a*).

The established cell lines retained the characteristics of the xenografts from which they were derived, which in turn resembled the patient tumours as far as could be determined (for example, in histological appearance and immunohistochemical staining, cell size and chromosomal complement). In addition, the radiation sensitivities of cells whether from xenografts or cultured cell lines derived from the same tumour did not differ significantly (Duchesne, unpublished observations), supporting the usefulness of this approach in establishing representative cell lines.

The characterisation of the lines with respect to expression of neural and epithelial markers confirms the current view that all types of lung carcinoma arise from an epithelial origin. Previous observations of the expression of neuroendocrine enzymes and peptides by small-cell carcinomas (e.g. Gazdar *et al.*, 1981*b*; Moody *et al.*, 1981; Sheppard *et al.*, 1984) demonstrated a clear distinction from the non-small-cell tumours, and suggested that these tumours might be neuroectodermal in origin. Later reports (e.g. Carney *et al.*, 1985), supported by the present findings, have demonstrated the expression of such properties by some non-small-cell carinomas, although generally to a lesser extent. This is in keeping with the concept that lung cancer cells may express markers of more than one differentiation pathway, and emphasises the close relationships of the cell types.

The pattern of intermediate filament expression in the different histological types also reflects the capacity of lung carcinoma cells to express markers of differing cell lineages, although neoplasms generally retain the intermediate filament structure of their tissue of origin (Altmannsberger *et al.*, 1981). All the lines studied here expressed cytokeratins, in keeping with previous reports (e.g. Bernal *et al.*, 1983; Blobel *et al.*, 1984). Staining for neurofilament was observed in all the small-cell carcinomas, both classical and variant, and adenocarcinomas, but not in the squamous or large-cell lines. The expression of neurofilaments in small-cell carcinomas is still debated (e.g. Bergh *et al.*, 1984; Van Muijen *et al.*, 1984), but the present observations would be consistent with the explanation offered by Clark *et al.* (1985), that neurofilament expression is facultative, and occurs only in terminally differentiated cells.

The interrelationships of the cell types are also revealed by the observed alterations in morphological and biochemical characteristics of the lines through successive passages, illustrated by the changing characteristics of HX148/148M and HX149/149M. The emergence of a large-cell component in the cell line of HX148 was associated with the appearance of a bimodal chromosomal distribution (the higher modal number being distinct from that of any of the other tumour lines) and a change in the morphology of some of the cells and in radiation sensitivity. It was not possible to determine whether the large-cell component had been present initially, but further studies are underway to clone the two cell populations, and investigate the change in characteristics further. The change in growth pattern and cell type between HX149 and HX149M occurred in xenograft passage, and has not been observed in the cultured line. This may indicate modification of the tumour cell type by the host environment as previously suggested by Carney *et al.* (1983), or the existence of a small initial subpopulation of cells particularly suited to growth in this host which overgrew the original cell type. The observation is of clinical relevance as it may be analogous to the changes in morphology seen in small-cell tumours after therapy, and provides a reason for possible therapeutic resistance in what is usually considered a sensitive tumour.

No consistent differences in biological behaviour or tumour markers were found between tumours from primary or metastatic sites, although it is recognised that the number of lines is small, and that any such differences might therefore not be detected. Consistent differences in radiation sensitivity have been found, however, between the different histological types, with the lines with a large-cell component exhibiting intrinsic radioresistance, and the other histological types being relatively radiosensitive. There is a suggestion that those small-cell lines which had not been previously irradiated (e.g. HX168, HC38 and HC39) were more radiosensitive than those which had been heavily pretreated, such as HC12 which showed therapeutic resistance to both drugs and radiation in the patient. Interestingly HC12, which is morphologically a classical small-cell carcinoma, exhibits a biochemical profile and radiation sensitivity intermediate between that of the other classical lines and the large-cell lines. It is tempting to speculate that the spectrum of radiosensitivity observed relates directly to the degree of expression of the neuroendocrine phenotype, and that the resistance to radiation observed clinically in some tumours may result from the presence or emergence of a large-cell, non-endocrine component.

The results presented here confirm the overlap of neuroendocrine and endodermal features in the small-cell and non-small-cell tumours and are in keeping with the current concept that all the tumours arise from a common cell of origin. It is suggested that there is no rigid distinction between the different tumour types but rather that the cells may undergo phenotypic alterations from one type to another. Radiation resistance appears to be related to the large-cell phenotype, and a spectrum of response is seen which parallels the spectrum of neuroendocrine expression.

The study would not have been possible without the co-operation of the clinicians from whose patients the specimens were obtained, in particular, Dr J. Yarnold, Dr I. Smith and Mr N. Wright. The authors are grateful to Dr P. Monaghan and Mr D. Roberts for performing and interpreting the electron microscopy. We are also indebted to Dr J. Millar, Dr V. Macaulay and Dr J. Delic for their collaboration with the radioassays, and to Dr G. Steel for his support.

### References

ALTMANNSBERGER, M., OSBORN, M., SCHAUER, A. & WEBER, K. (1981). Antibodies to different intermediate filament proteins. Cell type-specific markers on paraffin-embedded human tissues. *Lab. Invest.*, **45**, 427.

BEAVEN, M., WILCOX, G. & KERPSTRA, G. (1978). A microprocedure for the measurement of $^{14}CO_2$ release from [$^{14}C$] carboxyl-labelled amino acids. *Anal. Biochem.*, **84**, 638.

BERGH, J., NILSSON, K., DAHL, D., ANDERSSON, L., VIRTANEN, I. & LEHTO, V.-P. (1984). Expression of intermediate filaments in established human lung cancer cell lines. *Lab. Invest.*, **51**, 307.

BERNAL, S., BAYLIN, S., SHAPER, J., GAZDAR, A. & BOCHEN, L. (1983). Cytoskeleton-associated proteins of human lung cancer cells. *Cancer Res.*, **43**, 1798.

BLOBEL, G., MOLL, R., FRANKE, W. & VOGT-MOYKOPF, I. (1984). Cytokeratins in normal lung and lung carcinomas 1. Adenocarcinomas, squamous cell carcinomas and cultured cell lines. *Virch. Arch. (Cell Pathol.)*, **45**, 407.

BRADFORD, M. (1976). A rapid and sensitive method for the quantitation of microgram quantities of protein using the principle of protein-dye binding. *Anal. Biochem.*, **72**, 248.

CARNEY, D., BRODER, L., EDELSTEIN, M. & 6 others. (1983). Experimental studies of the biology of human small cell lung cancer. *Cancer Treat. Rep.*, **67**, 27.

CARNEY, D., GAZDAR, A., BEPLER, G. & 5 others. (1985). Establishment and identification of small cell lung cancer cell lines having classic and variant features. *Cancer Res.*, **45**, 2913.

CHEN, T.R. (1977). *In situ* detection of mycoplasma contamination in cell cultures by fluorescent Hoechst 33258 stain. *Exp. Cell. Res.*, **104**, 225.

CLARK, R., MIETTINEN, M., DE LEIJ, L. & DAMJANOV, I. (1985). Terminally differentiated derivatives of pulmonary small cell carcinomas may contain neurofilaments. *Lab. Invest.*, **53**, 243.

COOPER, E., SPLINTER, T., BROWN, D., MUERS, M., PEAKE, M. & PEARSON, S. (1985). Evaluation of a radioimmunoassay for neuron specific enolase in small cell lung cancer. *Br. J. Cancer*, **52**, 333.

COURTENAY, D. & MILLS, J. (1978). An *in vitro* colony assay for human tumours grown in immune-suppressed mice and treated *in vivo* with cytotoxic agents. *Br. J. Cancer*, **37**, 261.

DEACON, J., PECKHAM, M. & STEEL, G. (1984). The radioresponsiveness of human tumours and the initial slope of the cell survival curve. *Radiother. Oncol.*, **2**, 317.

DUCHESNE, G., PEACOCK, J. & STEEL, G. (1986). The acute *in vitro* and *in vivo* radiosensitivity of human lung tumour lines. *Radiother. Oncol.*, **7**, 353.

FERTIL, B. & MALAISE, E.-P. (1981). Inherent cellular radiosensitivity as a basic concept for human tumour radiotherapy. *Int. J. Radiat. Oncol. Biol. Phys.*, **7**, 621.

GAZDAR, A., CARNEY, D. & SIMS, H. (1981*a*). Heterotransplantation of small-cell carcinomas of the lung into nude mice: Comparison of intracranial and subcutaneous routes. *Int. J. Cancer*, **28**, 777.

GAZDAR, A., ZWEIG, M., CARNEY, D., VAN STEIRTEGHEN, A., BAYLIN, S. & MINNA, J. (1981*b*). Levels of creatine kinase and its BB isoenzyme in lung cancer specimens and cultures. *Cancer Res.*, **41**, 2773.

GAZDAR, A., CARNEY, D., NAU, M. & MINNA, J. (1985). Characterisation of variant subclasses of cell lines derived from small cell lung cancer having distinctive biochemical, morphological and growth properties. *Cancer Res.*, **45**, 2924.

MAKIN, C., BOBROW, L. & BODMER, W. (1984). Monoclonal antibody to cytokeratin for use in routine histopathology. *J. Clin. Pathol.*, **37**, 975.

MILLAR, B., FIELDEN, E. & MILLAR, J. (1978). Interpretation of survival-curve data for Chinese hamster cells, line V79, using the multitarget, multitarget with initial slope and alpha-beta equations. *Int. J. Radiat. Biol.*, **33**, 599.

MOODY, T., PERT, C., GAZDAR, A., CARNEY, D. & MINNA, J. (1981). High levels of intracellular bombesin characterise human small-cell lung cancer. *Science*, **214**, 1246.

OBOSHI, S., TSUGAWA, S. & SEIDO, T. (1971). A new floating cell line derived from human pulmonary carcinoma of oat cell type. *Gann*, **62**, 505.

SHEPPARD, J., CORRIN, B., BENNETT, M., MARANGOS, P., BLOOM, S. & POLAK, J. (1984). Immunocytochemical localisation of neuron specific enolase in small cell carcinomas and carcinoid tumours of the lung. *Histopath.*, **8**, 171.

SIMMS, E., GAZDAR, A., ABRAMS, P. & MINNA, J. (1980). Growth of human small cell (oat cell) carcinoma of the lung in serum-free growth factor-supplemented medium. *Cancer Res.*, **40**, 4356.

VAN MUIJEN, G., RUITER, D., VAN LEEUWEN, C., PRINS, F. REITSEMA, K. & WARNAAR, C. (1984). Cytokeratin and neurofilament in lung carcinomas. *Am. J. Pathol.*, **116**, 363.

WORLD HEALTH ORGANISATION (1981). Histological classification of lung tumours. In *International Histological Classification of Tumours: Histological Typing of Lung Tumours*. Second edition. WHO: Geneva.